# Detection of Multiple Human Viruses, including Mpox, Using a Wastewater Surveillance Approach in Brazil

**DOI:** 10.3390/pathogens13070589

**Published:** 2024-07-16

**Authors:** Juliana Calabria de Araujo, Ana Paula Assad Carvalho, Cintia D. Leal, Manuelle Natividade, Marcus Borin, Augusto Guerra, Natália Carobin, Adriano Sabino, Mariana Almada, Maria Cristina M. Costa, Flavia Saia, Livia V. Frutuoso, Felipe C. M. Iani, Talita Adelino, Vagner Fonseca, Marta Giovanetti, Luiz Carlos Junior Alcantara

**Affiliations:** 1 Department of Sanitary and Environmental Engineering, School of Engineering, Universidade Federal de Minas Gerais—UFMG, Belo Horizonte 31270901, Brazil; 2 Department of Social Pharmacy, College of Pharmacy, Federal University of Minas Gerais, Belo Horizonte 31270901, Brazil; 3 Clinical and Toxicological Analysis Department, College of Pharmacy, Federal University of Minas Gerais, Belo Horizonte 31270901, Brazil; 4 Centro Federal de Educação Tecnológica—CEFET-MG, Belo Horizonte 30421169, Brazil; 5 Department of Marine Sciences, Marine Institute, Universidade Federal de São Paulo—UNIFESP, Baixada Santista 11070100, Brazil; 6 General Coordination of Arbovirus Surveillance, Department of Health and Environmental Surveillance, Ministry of Health, Brasília 70304000, Brazil; 7 Central Public Health Laboratory, Fundação Ezequiel Dias, Belo Horizonte 30510010, Brazil; felipe.iani@funed.mg.gov.br (F.C.M.I.); talita.adelino@funed.mg.gov.br (T.A.); 8 Coordination of Surveillance, Emergency Preparedness and Response (PHE), Pan American Health Organization/World Health Organization (PAHO/WHO), Pan-Americana da Saúde/Organização Mundial da Saúde (OPAS/OMS), Brasilia 70312970, Brazil; vagner.fonseca@gmail.com; 9 Department of Sciences and Technologies for Sustainable Development and One Health, Universita Campus Bio-Medico di, 00128 Roma, Italy; giovanetti.marta@gmail.com; 10 René Rachou Institute, Fundação Oswaldo Cruz, Belo Horizonte 30190001, Brazil; alcantaraluiz42@gmail.com

**Keywords:** Mpox virus, genomic surveillance, wastewater surveillance, influent wastewater treatment plants (WWTP), whole-genome sequencing, sewage virome, qPCR

## Abstract

Sewage surveillance can be used as an effective complementary tool for detecting pathogens in local communities, providing insights into emerging threats and aiding in the monitoring of outbreaks. In this study using qPCR and whole genomic sewage surveillance, we detected the Mpox virus along with other viruses, in municipal and hospital wastewaters in Belo Horizonte, Brazil over a 9-month period (from July 2022 until March 2023). MPXV DNA detection rates varied in our study, with 19.6% (11 out of 56 samples) detected through the hybrid capture method of whole-genome sequencing and 20% (12 out of 60 samples) through qPCR. In hospital wastewaters, the detection rate was higher, at 40% (12 out of 30 samples) compared to 13.3% (4 out of 30 samples) in municipal wastewaters. This variation could be attributed to the relatively low number of MPXV cases reported in the city, which ranged from 106 to 341 cases during the study period, and the dilution effects, given that each of the two wastewater treatment plants (WWTP) investigated serves approximately 1.1 million inhabitants. Additionally, nine other virus families were identified in both hospitals and municipal wastewaters, including Adenoviridade, Astroviridae, Caliciviridae, Picornaviridade, Polyomaviridae, Coronaviridae (which includes SARS-CoV-2), Herspesviridae, Papillomaviridae and Flaviviridae (notably including Dengue). These findings underscore the potential of genomic sewage surveillance as a robust public health tool for monitoring a wide range of viruses circulating in both community and hospitals environments, including MPXV.

## 1. Introduction

The Mpox virus (MPXV) is a double-stranded enveloped DNA virus, belonging to the *Poxvirus* family, genus *Orthopoxvirus*, and is related to the variola virus, the causative agent of smallpox [1]. The disease caused by the Mpox virus is a zoonotic infectious disease that transmits between animals and humans, and the risk factors include close and sexual contact with an infected person, contact with fomites, infected animals or aerosolized infectious material [1].

In May 2022, an Mpox epidemic outside of Africa gained international attention. On 23 July 2022, the World Health Organization declared the Mpox epidemic a public health emergency of international concern, and on 29 July 2022, the Brazilian Ministry of Health established the Center of National Public Health Emergency Operations for Mpox (COE-MPOX) to coordinate the country’s public health responses to the disease [2].

In Brazil, the first Mpox confirmed case was reported on 9 June in the state of São Paulo, and on 15 June, Brazil had five confirmed cases according to the Ministry of Health [2]. The first fatal case in the world was reported on 28 July in Belo Horizonte (state of Minas Gerais), Southeast Brazil [3]. On 18 August, the municipal health secretariat of Belo Horizonte reported 106 confirmed and 14 probable cases in its first report (First Monkeypox report, Prefeitura de Belo Horizonte, 2022) [4,5]. From 1 June 2022 to 28 February 2023, Brazil recorded 10,656 confirmed and probable Mpox cases, according to the 20th Epidemiological Bulletin of Monkeypox by the COE—Ministério da Saúde [2]. In March 2023, when the Ministry of Health initiated Mpox vaccinations, prioritizing high-risk groups such as people living with HIV/AIDS with an immune status identified by the CD4 T lymphocyte cells count of less than 200 in the last six months, and health professionals who work directly with the virus in laboratories (pre-exposure). At this time, Brazil had reported 52,109 suspected cases, of which 10,378 (19.9%) were confirmed [6].

Sewage surveillance has been recognized as a valuable method for monitoring pathogen circulation at the community level. Its effectiveness was demonstrated during the COVID-19 pandemic, which enabled the detection of SARS-CoV-2 by tracking the virus in sewage samples from numerous cities worldwide [7,8,9], including the city of Belo Horizonte [10]. Previous studies have detected MPXV in sewage samples in the Netherlands, Rome and Paris [11,12,13], as the virus is excreted into wastewater through skin lesions, nasal secretion, urine, and feces [14]. In our research, we investigated the circulation of MPXV (and other viruses) in sewage samples collected in Belo Horizonte using whole-genome sequencing. Belo Horizonte is a largest Brazilian city from the state of Minas Gerais, with a population around 2.7 million people and with a metropolitan area of 6 million people [15]. This study demonstrated the potential of genomic sewage surveillance for the early detection of emerging and reemerging pathogens in the community for aiding in outbreak response.

## 2. Material and Methods

Sewage samples were collected from four locations in the metropolitan area of Belo Horizonte: two hospitals (Hospital A, the reference hospital for infectious disease treatment in the state of Minas Gerais, and Hospital B, a general practice hospital aiding pregnant women and high-risk prenatal care) and two municipal wastewater treatment plants (WWTP), which, together, process sewage from approximately 2.2 million people. The mean influent loads are 172,000 m^3^ per day for WWTP-A and 142,200 m^3^/day for WWTP-B. The geographic coordinates for these locations are WWTP-A: 19°54′20.2″ S 43°53′15.5″ W, WWTP-B: 19°49′22.5″ S 43°53′44.2″ W, Hospital A: 19°59′13.5″ S 43°59′12.1″ W, and hospital B: 19°49′03.0″ S 43°56′53.4″ W.

Sewage samples were collected bi-monthly from 23 July 2022 to 15 March 2023. In total, 60 samples were collected: 30 from the two hospitals (15 samples each) and 30 from the municipal WWTPs (16 samples from WWTP-A and 14 from WWTP-B). However, 13 samples from each hospital and 30 samples from the WWTPs A + B were sequenced (totaling 56 samples).

Hospital sewage samples were collected as described previously [16]. For the WWTPs, 24 h composite samples were collected at the entrance and filtered through electronegative membranes (30 to 50 mL) [10], with slight modifications from previous methods: MgCl_2_ (2.5 M) was not added and the pH was not adjusted to 3.5. Viral genetic material was extracted from the membranes using a commercial kit (AllPrep PowerViral DNA/RNA, Qiagen^®^, Hilden, Germany) according to the manufacturer’s instructions. The extracted genetic material was resuspended in 100 µL of ultrapure water (free of RNases) and stored at −80 °C.

Samples were tested for MPXV using a quantitative PCR (qPCR) with the Kit molecular 5PLEX OPVX/MPXV/VZV/RP (Bio-Manguinhos, Rio de Janeiro, Brazil, https://www.bio.fiocruz.br/images/bm-bul-174-01-r-sn---multiplex--.pdf (accessed on 5 July 2024) on the 7500 Real-Time PCR System, following the manufacturer’s instructions. The tests targeted the OPG068 gene for MPXV, ORF38/UL21 protein for Varicella Zoster virus (VZV), the Varicella Zoster Virus (VZV), MC001 protein region for Molluscum Contagiosum Virus (MOCV), and putative 17 L protein from the OPG083 gene for the other orthopoxviruses (OPVs) [17]. The amplification conditions were 95 °C for 5 min; 40 cycles of 95 °C for 20 s followed by 60 °C for 30 s. The samples were also tested for Influenza A, Influenza B, and SARS-CoV-2 with the multi PLEX assay diagnostic Kit from Bio-Manguinhos, following the manufacturer’s instructions. The RT-qPCR assays for the detection of INFA/INFB/SC2 were performed according to the protocol developed by the Bio-Manguinhos Molecular Kit in a QuantStudio^®^7 Real-time PCR System (Thermo Fisher, Waltham, MA, USA). The amplification conditions were as follows: 50 °C for 15 min; 95 °C for 2 min, then 40 cycles of 95 °C for 20 s followed by 58 °C for 30 s. Since these kits are commercial, the manufacturer does not provide the sequences of the primers.

In total, 56 samples underwent whole-genome sequencing through target enrichment using a hybrid capture method. Target enrichment involves capturing the egenomic regions of interest by using biotinylated probes that hybridize to the target sequences. Probes are isolated using magnetic pulldown, selectively enriched for the desired regions. The target enrichment sequencing utilized the Illumina VSP panel, which characterizes 66 viruses, including DNA and RNA viruses such as Polyomavirus, HPV, Mpox, Poliovirus, Influenza, SARS-CoV-2, among others (“https://www.illumina.com/products/by-type/sequencing-kits/library-prep-kits/viral-surveillance-panel.html” (accessed on 5 July 2024)).

Sequencing libraries were prepared by first synthesizing cDNA from concentrated wastewater samples. This process employed the Illumina RNA Prep with Enrichment Indexes Set A (for 96 samples) (Illumina, San Diego, CA, USA) and the VSP (Illumina). Libraries were sequenced on the NextSeq™ 2000 System with a read length of 2 × 150 bp and a sequencing. FASTQ raw sequencing data were analyzed using the Illumina DRAGEN™ Microbial enrichment pipeline (available on the BaseSpace™ Sequence Hub) for viral detection using default parameters. Raw data were also analyzed using Genome Detective software 2.48 [18]. The sequences were deposited in GenBank under sample codes SAMN42174356 to SAMN42174437.

## 3. Results and Discussion

MPXV DNA was detected via hybrid capture method for whole-genome sequencing (WGS) and confirmed using qPCR in 10/15 (66.7%) samples from Hospital A, spanning from 23 July 2022 to 15 March 2023 (Table 1). In Hospital B, two out of 13 samples (15.4%) tested positive for MPXV. In municipal wastewaters, MPXV DNA was detected in 4 out of 30 samples (13.3%) using both methods of detection (WGS and qPCR, Table 1).

The higher frequency of detection in the sewage samples from Hospital A (66.7%) can be attributed to its role as the reference hospital for infectious disease treatment in the state of Minas Gerais, which serves patients from many cities, including Belo Horizonte. Notably, the first fatal case reported in Brazil, on 28 July 2022, was a patient being treated at Hospital A, where this study was conducted (see Table 1, sewage from Hospital A was positive on 23 July). Furthermore, hospital sewage samples were collected directly from the hospital manhole before the sewage was discharged in the sewer network, thus avoiding any dilution.

Overall, MPXV DNA was detected in 16/60 (26.6%) of the total sewage samples combining both methods of detection (qPCR and WGS).

The detection frequency of MPXV DNA in municipal sewage observed (13.3% in WWTP A and B, 4 out of 30 samples) could be attributed to the limited number of MPXV cases reported in the city (as shown in Table 1): 106 confirmed cases on 18 August 2022, 324 on 30 November 2022, 339 on 11 January 2023 [19], and 8 new cases (2 confirmed and 6 suspected) on 24 February 2023 [20].

Additionally, the dilution factor should be considered, as each WWTP serves a population of approximately 1.1 million people. A previous study in the Netherlands [11] reported the detection frequency of MPXV DNA using qPCR in sewage samples from Amsterdam city districts and Schiphol airport, ranging from 14% to 62.5%, when the national case count was 1087, with most cases located in Amsterdam. The frequency of detection varied due to the sampling location and the number of inhabitants connected to the WWTPs investigated (which varied from 16,000 to 648,000 inhabitants in Amsterdam West) [11]. The detection frequency reported in Schiphol airport sewage was 26.1% (6/23) for samples collected from 17 May to 3 July 2022 [11]. Another study in Italy reported a detection frequency of 15% (3 out of 20 sewage samples of Rome airport, from 30 May to 3 August 2022) [12] when the number of cases in the country was 20 (by May 2022). In Paris, a previous study reported positive correlation between frequency of detection and cumulative cases, showing detection of 20% when the number of cumulative cases was 100 and about 30% when the number of cumulative cases was 300 and weekly new cases about 100 [13]. Therefore, the detection frequency values observed in the present study (ranging from 13% at the influent of WWTPs and 67% at hospital A, Table 1) are in line with previous works and reflect the locations sampled and the epidemiological situation observed in the city of Belo Horizonte.

This is the first comprehensive report of multiple virus detection, including MPXV DNA, in sewage in Brazil, from an area with relatively few cases (339 cumulative cases from August to January 2023, as shown Table 1). In Brazil, as reported by the Ministry of Health up to 31 March 2023, the proportion of confirmed Mpox cases was 19.9% (10,378 out of 52,109 notified suspected cases) with an additional 330 cases (0.6% out of 52,109 notified cases) classified as probable (according to the 21st Epidemiological Bulletin of Monkeypox by the COE—Ministério da Saúde) [6]. These findings underscore the utility of wastewater surveillance as an invaluable epidemiological tool for monitoring emerging infectious diseases, highlighting the importance of employing both detection methods (WGS and qPCR) and extending surveillance to multiple sampling locations, including hospitals and municipal WWTPs.

**Figure 1:**
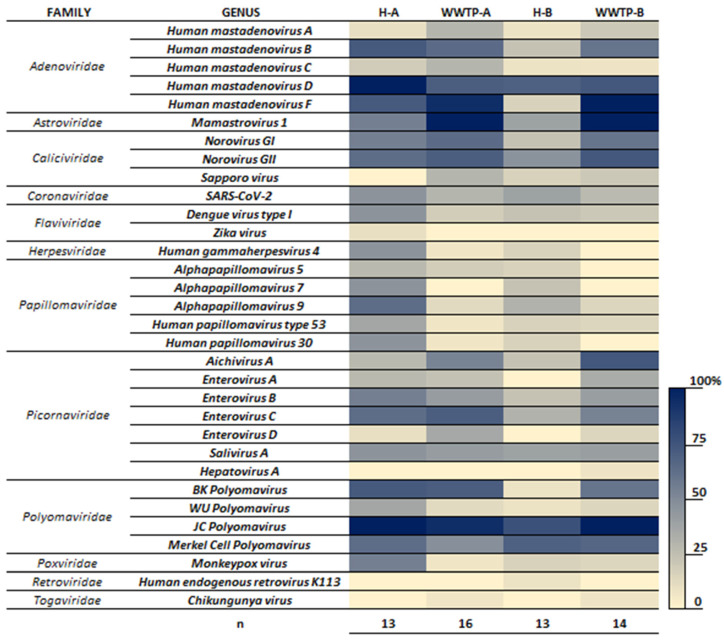
Heatmap displaying the viruses detected in wastewater samples from various locations using the VSP panel and whole-genome sequencing. The scale indicates the frequency of virus detection in the total samples from each location. H-A represents Hospital A (13 samples), H-B represents Hospital B (13 samples), WWTP-A (16 samples), and WWTP-B (14 samples).

In addition to the Mpox virus, nine other virus families were identified in samples from Hospital A and municipal WWTPs (Figure 1). These include Adenoviridade, Astroviridae, Caliciviridae, Picornaviridade, Polyomaviridae, Coronaviridae (SARS-CoV-2), Herspesviridae, Papillomaviridae and Flaviviridae (Figure 1). Members of these families represent some of the most significant excreted human pathogens known to be shed in human waste and have previously been identified as part of the sewage virome [21], with the exception of Mpox and Dengue viruses.

Human Adenovirus and JC Polyomavirus, detected in over 80% of samples from both hospitals and WWTPs (Figure 1), are commonly used as fecal indicators due to their prevalence in sewage and their correlation with fecal pollution [21].

Figure 1 also shows the more prevalent viruses in hospital wastewaters, including species from Adenoviridae (human mastadenovirus B, D and F), Astroviridae (Mamastrovirus 1), Caliciviridae (Norovirus GI and GII), Picornaviridade (Enterovirus B, C and Salivirus A), Herpesviridae (Human gammaherpesvirus 4), Papillomaviridae (Alphapapillomavirus and Human papillomavirus), and Flaviviridae (Dengue virus type I).

Most viruses discussed in this study cause gastroenteritis in humans [22], such as the families Adenoviridae, Astroviridae, Caliciviridae, and Picornaviridae. Hospital A, which treats patients with HIV and other infectious diseases, frequently encounters diarrhea among these patients. Polyomaviridae, such as BK Polyomavirus and JC Polyomavirus, are widely distributed across global populations [23]. Typically, the primary infection of these viruses is asymptomatic and latent, becoming active due to immunosuppression. Previous studies have detected these viruses in urine samples from both HIV-positive patients and HIV-negative individuals who are immunocompromised [24]. Human papillomavirus (HPV) detected includes high-risk HPV types which are implicated in the development of cervical cancer [25] and are the most prevalent pathogens responsible for female cancers, particularly in developing countries [26].

Enterovirus causes severe respiratory illness in children and association between EV-D68 and neurological disease such as acute flaccid paralysis has been reported [27]. Wu Polyomavirus is commonly found in the respiratory tract samples of immunocompromised patients [28].

Concerning RNA viruses that cause respiratory infections, such as SARS-CoV-2 and Influenza A and B, the results are detailed in Table 2. Notably, SARS-CoV-2 RNA was detected in about 53% of the sewage samples tested by RT-PCR and 68% by WGS. The number of COVID-19 confirmed cases reported in Belo Horizonte during the investigated period were as follows: 4000 (September 2022), 6000 (November 2022), 14,000 (December 2022), 1000 in February, and 1000 in March 2023 (according to Prefeitura de Belo Horizonte, 2023) [29]. Influenza A RNA was detected only by RT-PCR (21.4% as shown in Table 2) and not by hybrid capture and WGS, indicating a sensitivity difference between the two methods. There was no detection of Influenza B RNA by either method, suggesting that this virus was not circulating in the investigated period or the prevalence was too low. In fact, the prevalence of Influenza B in Brazil during the investigated period was 0.2% of positive cases for respiratory viruses (August 2022), 1.2% (September 2022), 0.6% (October 2022), 0.3% (November 2022), 0.1% (December 2022), 0.7% (January 2023), 1.6% (February 2023) and 3.0% (March 2023) (according to the Respiratory diseases Information Bulletins of Fundação Oswaldo Cruz) [30].

In addition, it is also important to mention that by using the WGS, sequences of Dengue, Chikungunya, and Zika viruses were detected in some of these samples (Figure 1). For Dengue virus, the detection occurred in 8 out of 26 samples of hospital sewage and in 6 out of 30 samples from WWTPs A and B (in samples collected in February and March of 2023). Zika and Chikungunya sequences were detected in only one sample, respectively, in Hospital A sewage collected in 2 August 2023 and sewage from WWTP-A collected in 3 January 2023. The number of Dengue confirmed cases was 776 and 2874 in February and March of 2023 in the municipality of Belo Horizonte [31]. While there was only one case reported of Zika virus in Belo Horizonte in 2023, for Chikungunya, it was reported 404 and 1314 confirmed cases, respectively in February and March of 2023 [31]. A previous study [32] had mentioned the potential and challenges of using sewage surveillance to monitor human arboviral diseases but did not show any work that had in fact detected these viruses in wastewater samples.

To our knowledge, this is the first study that has applied the hybrid-capture target enrichment method and WGS to the successful detection and genetic characterization of multiple viruses in wastewaters during the Mpox outbreak in Brazil. This could provide crucial insights for viral surveillance and support public health authorities in guiding control actions.

## 4. Conclusions

In conclusion, this study underscores the effectiveness of genomic sewage surveillance, utilizing the hybrid-capture method and whole-genome sequencing (WGS), as a powerful tool for monitoring viruses in both community and hospital settings, including MPXV. Additionally, the study demonstrated that the qPCR diagnostic kit used for clinical samples (five PLEX assays diagnostic kit from Bio-Manguinhos), which is standard across Brazilian public health laboratories during the Mpox outbreak, also performed exceptionally well for monitoring wastewater samples, exhibiting a higher frequency of detection compared to WGS. This suggests that wastewater surveillance for Mpox and other viruses could be effectively conducted by central public health laboratories and should encompass both hospital and municipal wastewater systems. This approach could serve as a complement to clinical case studies, providing critical insights that help guide public health responses effectively.

## Figures and Tables

**Table 1:** Results of MPXV detection using qPCR and whole-genome sequencing and the number of Mpox confirmed cases reported in the municipality of Belo Horizonte. Samples were processed using a viral surveillance panel, sequenced on the NextSeq 2000 system, and analyzed with Genome Detective software.

Sample/Date	Number of Reads	Coverage %	qPCR Result/Cq Value	Number of Mpox Cases *
Hospital A (07/23/2022)	n.d.	n.d.	+ (32.0)	No report
Hospital A (08/12/2022)	16	0.16	+ (35.0)	106
Hospital A (09/01/2022)	n.d.	n.d.	+ (33.0)	159
Hospital A (09/21/2022)	n.d.	n.d.	n.d.	260
Hospital A (10/05/2022)	n.d.	n.d.	n.d.	287
Hospital A (10/19/2022)	n.s.	n.s.	+(35.0)	300
Hospital A (11/18/2022)	n.d.	n.d.	n.d.	311
Hospital A (11/30/2022)	340,702	100	+(18.8)	324
Hospital A (12/14/2022)	21,324	43.3	+(26.6)	333
Hospital A (01/11/2023)	44,367	82.4	+(29.0)	339
Hospital A (01/25/2023)	3811	9.9	+(32.0)	01
Hospital A (02/08/2023)	n.d.	n.d.	n.d.	01
Hospital A (02/24/2023)	5616	0.56	+(36.0)	02
Hospital A (03/01/2023)	n.d.	n.d.	n.d.	No report
Hospital A (03/15/2023)	n.s.	n.s.	+(38.0)	No report
Detection	6/13 = 46.1%		10/15 = 66.7%	
Hospital B (07/23/2022)	n.s.	n.s.	n.d.	
Hospital B (08/12/2022)	1369	0.73	+(36.0)	106
Hospital B (09/01/2022)	n.d.	n.d.	n.d.	159
Hospital B (09/21/2022)	n.d.	n.d.	n.d.	260
Hospital B (10/05/2022)	n.d.	n.d.	n.d.	287
Hospital B (10/19/2022)	n.s.	n.s.	n.d.	300
Hospital B (11/18/2022)	n.d.	n.d.	n.d.	311
Hospital B (11/30/2022)	n.d.	n.d.	n.d.	324
Hospital B (12/14/2022)	n.d.	n.d.	n.d.	333
Hospital B (01/11/2023)	53	0.52	n.d.	339
Hospital B (01/25/2023)	n.d.	n.d.	n.d.	01
Hospital B (02/08/2023)	n.d.	n.d.	n.d.	01
Hospital B (02/24/2023)	n.d.	n.d.	n.d.	02
Hospital B (03/01/2023)	n.d.	n.d.	n.d.	No report
Hospital B (03/15/2023)	n.d.	n.d.	n.d.	No report
Detection	2/13 = 15.4%		1/15 = 6.6%	
WWTP-A (08/12/2022)	n.s.	n.s.	+(38.0)	106
WWTP-A (02/08/2023)	2	0.068	n.d.	01
WWTP-B (02/08/2023)	4	0.045	n.d.	01
WWTP-B (03/01/2023)	7	0.052	n.d.	No report
Total detection	11/56 = 19.6%		12/60 = 20%	

Hospital samples (15 from each were tested using qPCR, and 13 underwent sequencing). Samples from WWTP-A (16 samples) and WWTP-B (14 samples) were collected from 12 August 2022 to 15 March 2023. Only two samples from these locations tested positive, and these results are shown in the table with the respective dates. The abbreviations used are as follows: “n.d.” indicates no detection of Monkeypox DNA by testing with qPCR and WGS using the VSP panel, and “n.s.” stands for not sequenced. * Number of Mpox confirmed cases in the municipality of Belo Horizonte, as reported by “Mpox | Prefeitura de Belo Horizonte (pbh.gov.br)” (accessed on 3 June 2024). From 25 January 2023, new cases were reported and not cumulative confirmed cases.

**Table 2:** Detection of SARS-CoV-2, Influenza A, and Influenza B in sewage samples from hospitals and WWTPs by applying hybrid capture-WGS with VSP and RT-PCR using BioManguinhos Kit *.

Sample/Date	WGS/SARS-CoV-2	SARS-CoV-2	Influenza A	Influenza B
Hospital A (07/23/2022)	1013 reads	+(32.0)	n.d.	n.d.
Hospital A (08/12/2022)	n.d.	n.d.	n.d.	n.d.
Hospital A (09/01/2022)	n.d.	n.d.	n.d.	n.d.
Hospital A (09/21/2022)	n.d.	+(31.9)	n.d.	n.d.
Hospital A (10/05/2022)	n.d.	+(31.0)	+(29.7)	n.d.
Hospital A (10/19/2022)	n.s.	n.d.	n.d.	n.d.
Hospital A (11/18/2022)	901 reads	+(28.3)	+(36.1)	n.d.
Hospital A (11/30/2022)	n.d.	+(28.0)	n.d.	n.d.
Hospital A (12/14/2022)	n.d.	n.d.	n.d.	n.d.
Hospital A (01/11/2023)	n.d.	+(33.4)	n.d.	n.d.
Hospital A (01/25/2023)	n.d.	n.d.	+(30.9)	n.d.
Hospital A (02/08/2023)	537 reads	n.d.	n.d.	n.d.
Hospital A (02/24/2023)	18 reads	+(30.9)	+(31.3)	n.d.
Hospital A (03/01/2023)	632 reads	+(31.9)	n.d.	n.d.
Hospital A (03/15/2023)	n.s.	+(32.3)	n.d.	n.d.
Hospital B (09/01/2022)	555 reads	n.d.	n.d.	n.d.
Hospital B(12/01/2022)	1261 reads	+(34.1)	n.d.	n.d.
Hospital B (02/07/2023)	381 reads	n.d.	n.d.	n.d.
Hospital B (02/23/2023)	1156 reads	+(33.6)	n.d.	n.d.
Hospital B (03/01/2023)	735 reads	n.d.	n.d.	n.d.
Hospital B (03/15/2023)	n.s.	+(30.6)	n.d.	n.d.
WWTP-A (02/08/2023)	433 reads	n.d.	+(31.6)	n.d.
WWTP-A (02/23/2023)	780 reads	+(35.3)	+(33.6)	n.d.
WWTP-A (03/01/2023)	94 reads	n.d.	n.d.	n.d.
WWTP-A (03/15/2023)	401 reads	+(34.8)	n.d.	n.d.
WWTP-B (02/23/2023)	1014 reads	+(34.7)	n.d.	n.d.
WWTP-B (03/01/2023)	1373 reads	n.d.	n.d.	n.d.
WWTP-B (03/15/2023)	50 reads	n.d.	n.d.	n.d.
Detection	17/25 = 68%	15/28 = 53.6%	6/28 = 21.4%	0%

* RT-qPCR assays for the detection of INFA/INFB/SC2 were performed according to the protocol developed by the Bio-Manguinhos Molecular Kit and instructions provided by the manufacturer in a QuantStudio^®^5 Real-time PCR System (Thermo Fisher). n.d. not detected. n.s. not sequenced. Influenza A and Influenza B genomes were not detected by hybrid capture-WGS, and the results are not shown in this table.

## Data Availability

The sequences were deposited in GenBank under sample codes SAMN42174356 to SAMN42174437.
